# Mitigating COVID-19 on a small-world network

**DOI:** 10.1038/s41598-021-99607-z

**Published:** 2021-10-14

**Authors:** Marvin Du

**Affiliations:** College of Chemistry, University of California, Berkeley, Berkeley, CA 94720 USA

**Keywords:** Epidemiology, Infectious diseases

## Abstract

Continuous deterministic models have been widely used to guide non-pharmaceutical interventions (NPIs) to combat the spread of the coronavirus disease 2019 (COVID-19). The validity of continuous deterministic models is questionable because they fail to incorporate two important characteristics of human society: high clustering and low degree of separation. A small-world network model is used to study the spread of COVID-19, thus providing more reliable information to provide guidance to mitigate it. Optimal timing of lockdown and reopening society is investigated so that intervention measures to combat COVID-19 can work more efficiently. Several important findings are listed as follows: travel restrictions should be implemented as soon as possible; if ‘flattening the curve’ is the purpose of the interventions, measures to reduce community transmission need not be very strict so that the lockdown can be sustainable; the fraction of the population that is susceptible, rather than the levels of daily new cases and deaths, is a better criterion to decide when to reopen society; and society can be safely reopened when the susceptible population is still as high as 70%, given that the basic reproduction number is 2.5. Results from small-world network models can be significantly different than those from continuous deterministic models, and the differences are mainly due to a major shortfall intrinsically embedded in the continuous deterministic models. As such, small-world network models provide meaningful improvements over continuous deterministic models and therefore should be used in the mathematical modeling of infection spread to guide the present COVID-19 interventions. For future epidemics, the present framework of mathematical modeling can be a better alternative to continuous deterministic models.

## Introduction

Many nations have been forced to take non-pharmaceutical interventions (NPIs), such as social distancing^1,2^, face mask wearing^3^, and even school and business closures, to combat the COVID-19 pandemic. Mathematical modeling has been used to provide vital information of how the virus spreads and thus affords viable means to provide guidance to develop sustainable mitigation strategies^4,5^. Most of the epidemiological modeling works appeared so far are based on continuous deterministic compartment models^6–9^. In those models, the population of interest is divided into compartments of susceptible, infected, and recovered. Variants of the SIR model are also widely used to study the spread of infectious diseases. Some exceptions to the continuous deterministic models do exist, such as those based on branching process networks^10^ and small-world networks^11^.

A flawed assumption underlying the continuous deterministic models is that the infection transmission is homogeneous, i.e., all susceptible people have equal chances to get infected by a given infectious person regardless of whether they have direct contact with the infected. But more fundamentally, continuous deterministic models fail to take into consideration the interactions among people in society. This drawback can inevitably diminish the validity of the modeling results. Studying the spread of infectious diseases on networks is the perfect remedy to the weakness of the continuous deterministic models^12^. There are two types of popular network models that can be employed: scale-free networks^13^ and small-world networks^14^. Both types of networks have a low degree of separation. Scale-free networks have the desirable power-law degree distribution, but their clustering coefficient is generally too low. On the other hand, small-world networks have a satisfactorily high clustering coefficient but an exponential degree distribution which may not characterize social networks well. It is true that scale-free networks can be modified to have a larger clustering coefficient but doing so may introduce some unwanted characteristics into the networks that social networks do not possess. An example is the work by Klemm and Eguiluz^15^. The model does have a power-law degree distribution and a large clustering coefficient, but some nodes in the network are connected to all, or almost all, other active nodes. This feature is certainly not a realistic representation of our society.

A recent study shows that social networks are at best weakly scale-free, and many of them are not scale-free at all^16^. This finding makes small-world networks more appealing than scale-free networks in the study of infection transmission since small-world networks have a large clustering coefficient, which is one of the most important characteristics of social networks. In my opinion, the clustering coefficient plays a bigger role than degree distribution in affecting how infections spread across networks. Therefore, in this study, small-world networks are chosen because they possess the two defining features of human society: a high clustering coefficient and a low degree of separation. Here, I study how COVID-19 spreads on a small-world network and how the spread responds to imposing and lifting mitigation measures intended to lessen the severity of the pandemic.

## Methods

The small-world network used in this work is a variant^17^ of the original Watts and Strogatz model^14^. The network model starts with a ring or lattice of N vertices (also referred to as nodes), each connected to its k neighbors on each side by regular edges (or links). Shortcut edges are then added between randomly chosen pairs of vertices with a probability p for each regular edge (e.g., p = 0.01 means that 0.01 k random or shortcut edges are added to each vertex). To make the network connected by random edges, $N\gg k\gg \mathrm{ln}(N)\gg 1$ must be satisfied^14^. In epidemiological modeling, an edge is a contact between two vertices that can potentially transmit infection. These regular edges represent the pathways for community transmissions which are a result of daily social interactions among people, while the long-range random edges represent the routes for infection transport across communities by way of travel^18^. It is apparent that on the small-world network community transmission is controlled by the regular edges and the imported cases by the random edges.

In the process of modeling the spread of infection, a vertex can have one of the following states: susceptible, infected but not yet infectious (i.e., latent), symptomatically infectious, asymptomatically infectious, recovered, and deceased. At the beginning of each realization, all vertices are assigned as susceptible except for just one vertex where the initial state is infectious (either symptomatically or asymptomatically). The process proceeds as follows: at each new time step, a susceptible vertex has a probability of ${R}_{0}/2k{T}_{i}$ to become infected by each infectious vertex that is connected by an edge (either regular or random) where R_0_ is the basic reproduction number, 2 k is the number of regular edges connecting each vertex to its neighboring vertices, and T_i_ is the duration (in terms of time steps or days) that an infectious vertex needs to recover. Upon infection, the vertex remains non-infectious during a latent period T_l_, and after this period, it becomes infectious, either symptomatically or asymptomatically. In the meantime, a symptomatically infectious vertex can die with a probability of R_mortality_ in the entire infectious period or recover if the duration of infection has reached T_i_. The asymptomatically infectious vertices do not die but remain infectious until they have been infectious for a period of T_i_. At the time a susceptible vertex becomes infected, it has a probability of R_asymp_ to be classified as asymptomatic and a probability of 1 − R_asymp_ as symptomatic. Since it has been reported that recovered patients can potentially get infected again^19^, re-infection is allowed for recovered vertices to become susceptible.

In the modeling, NPIs to reduce the spread of infection are achieved by reducing the number of regular edges that each vertex has and/or the number of random edges. The former resembles the reduction of community transmission accomplished by social distancing, face mask wearing, contact tracing, telecommuting, sheltering-in-home, etc.; the latter mirrors limiting travel as well as screening and mandatory quarantining at travel hubs.

Once the spread of the infection is under control, society needs to reopen since lockdown negatively impacts every person. On the network, reopening is realized by increasing the number of regular edges as well as random edges. In the real world, this is to alleviate the mitigation measures by allowing people to go back to school and work, dine in restaurants, resume routine medical appointments, and travel domestically and internationally, among other things.

It should be noted that in this and other epidemiological modeling, it is impossible to quantify in advance the effectiveness of each mitigation measure. For example, no one knows the exact effect of social distancing or wearing face masks all the time by a certain percentage of people. The only thing that can be certain is that taking these measures can potentially reduce transmission of the disease. As such, taking each measure to reduce community transmission is equivalent to reducing the number of regular edges. Limiting travel, screening travelers, and quarantining possibly infected travelers are equivalent to reducing long-range random edges. In this work, those traveler-related measures are represented by reducing the number of random edges.

The small-world network has been tested for a wide range of selections of N, k, and p to ensure the network possesses the desired high clustering and low degree of separation. Ideally, N should be selected as large as possible, but a larger N requires more computational resources. In this study, N is set to be 100,000. In the base-case network, each vertex has k = 100 regular edges on each side to connect it to its neighbors (representing people that an individual has routine and regular physical contact with, e.g., family members, co-workers, and friends) and a total of 100,000 random edges (i.e., on average, each vertex has one long-range shortcut connected to a remote vertex). Lockdown and reopening are implemented by changing the number of edges.

It should be pointed out that k and p are meaningful only in a relative sense. Choosing k = 100 and p = 0.01 for the base-case study does not imply that each person in society interacts with 200 people in daily activities and knows only one person in far-away areas. But rather, the changes from k = 100 to k = 50 or from p = 0.01 to p = 0.001 mean that, on average, each person reduces the daily infection-transmissible interactions by half and/or long-distance travel by 90%. Supplementary Fig. 1 shows that for different values of k and p, the general patterns of the model results are quite similar, and in particular, the final levels of total deaths and the fraction of population that remains susceptible to infection are essentially the same. This is a clear indication that the small-world network is robust for a wide range of k and p.

Another point that needs to be made is that in real-world social networks, different individuals have different extents of interactions with others and different capabilities of transmitting infections once infected. In this study, k and p are nevertheless assumed to be the same for all vertices because adequate data do not exist to assert any postulations of universal probability distributions of k and p, especially in the context of contact-networks for COVID-19 infection transmission. Furthermore, it is unknown how individuals with different levels of social interactions respond to social distancing and lockdown orders. Finally, there currently exists no small-world network model that allows variable k and p for different vertices. This limitation certainly deserves further investigation once adequate data are garnered and such a small-world network model is developed. Even with constant k and p, each individual, represented as a vertex, can still have the potential to infect different numbers of people because for any given infectious vertex, whether any linked vertices get infected and at which time step are randomly chosen.

Unless clearly stated otherwise, for each case the modeling is done with 100 realizations. For all the cases modeled, it seems that this is adequate to produce stable statistics. Sensitivity tests were conducted to make sure that more realizations in each modeled scenario and changing the location of the seed node did not change the modeling results.

Several epidemiological parameters are needed in the present model. A wide range of the basic reproduction number R_0_ has been reported. One of the earliest reports from Wuhan, China finds it in the range of 2.24 ~ 3.58^18^. In the period between February 25 and March 12, 2020, R_0_ values associated with the outbreak in Italy were in the range of 2.43 ~ 3.10^20^. Much larger values were also reported^21^. Here I set it to be 2.5 in line with recent continuous deterministic modeling studies^4,5^. Similarly, the number of days an infected individual remains infectious (before recovery) is highly variable^22–24^. Duration of recovery was taken to be 15 days, which was divided into a latent period T_l_ and infectious period T_i_. Furthermore, each infectious vertex is assumed to be of the same infectiousness regardless of whether it is infected by symptomatically infectious or asymptomatically infectious vertices. In the modeling, the average latent period was taken to be 3 days^25^ and the average infectious period was taken to be 12 days. Among those infected, 27% to 57% could be asymptomatic^26–28^, so in this work, I set ${R}_{asymp}=0.3$. The mortality rate, R_mortality_, is also highly uncertain as in certain regions it was high but substantially lower in other regions. Without loss of generality, I took it to be 0.05 since it accounts for only a very small fraction of infections, so it barely changes the dynamics of the spread of disease on the network. Finally, based on epidemiological data it was assumed that a recovered vertex had a probability of 20% to get reinfected in 180 days^19^. In the modeling, some degree of randomness is introduced by allowing the latent period to vary between 0 and 2T_l_, and the infectious period to vary between 0.5T_i_ and 1.5 T_i_.

In this study, NPIs are achieved by reducing the number of regular and random edges. The response of infection transmission to the intervention measures is complex and some features are quite difficult to be quantified. One of the limitations in this study is the assumption that the symptomatically and asymptomatically infected have the same response to the mitigation measures. There exists evidence^28^ that asymptomatic and even pre-symptomatic patients behave differently from those who are symptomatically infectious. In the present work, symptomatic and asymptomatic patients are, for simplicity, treated in the same way in terms of their capability of transmitting the infection. The effects of this over-simplification should be explored once adequate epidemiological data become available.

To reinforce the argument that in this study the values of k and p themselves are not important (as long as they are in the right ranges to keep the network well-connected), the results of a sensitivity test are presented in Supplementary Fig. 2. It shows the remarkable similarity of how the network responds to a 25% reduction of k values for two different base cases: k = 100 and k = 75. This suggests that the lack of knowledge of some of the network parameters does not affect our ability to examine the effectiveness of the NPI measures.

## Results

### Base case study

**Figure 1:**
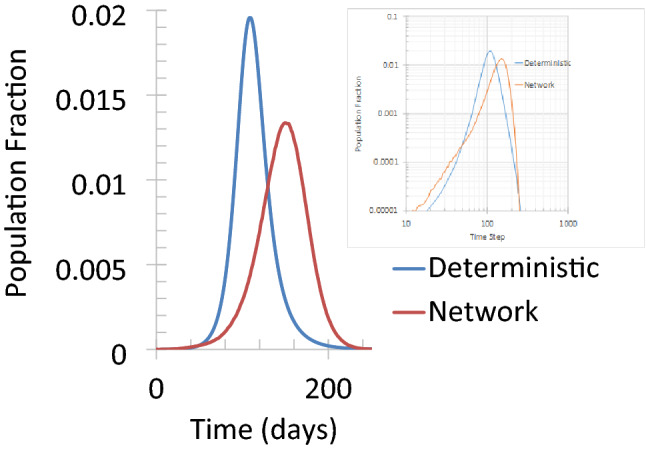
A comparison of results from a deterministic SIR model and a small-world network model. For the network model, in 9 out of 100 realizations the infection died out after at most one vertex was infected by the seed. The curve for the network represents the average of the remaining realizations that can last until herd immunity is achieved. The inset shows the evolution of infection spread on a log–log scale so that the exponential growth can be clearly revealed.

A case study was carried out to compare how different the modeling results are between the small-world network and continuous deterministic model. For simplicity in this comparison, both R_mortality_ and R_asymp_ were set to be 0, the latent period and infectious period were assumed to be 0 days and 15 days, respectively, and reinfection rate was set to be 0. For the final population fraction of susceptible and recovered, both models gave almost identical results. However, the numbers of daily new cases are quite different, as shown in Fig. 1.

Figure 1 shows that the spread of the disease can be significantly different between a continuous deterministic model and a small-world network model. The overall spread is much slower on the network, suggesting that the way the infection spreads can profoundly change the outcome. More specifically, the first major difference is that in the continuous deterministic model, the infection can sustain itself until herd immunity is achieved if ${R}_{0}\ge 1$. However, on the network, there is a significant chance that the infection dies out very quickly: with R_0_ = 2.5, 2.0 and 1.5, in 12, 18 and 53 out of 100 realizations the virus can only spread to just a few (less than 10) vertices, respectively. Secondly, in both models, the growth of new cases is piecewise exponential but is faster in the deterministic model. A remark that needs to be made here is that the exponential growth seems to be universal; therefore, it is not necessarily a warning sign if clinical/epidemiological evidence reveals exponential growth. Thirdly, the peak shows up much later and at a lower level in the network model than the prediction of the deterministic model, suggesting that the infection spread may be slower and less severe than what is predicted by the continuous deterministic models. This means that more time is available for public health agencies to prepare to “flatten the curve.”

The sensitivity of modeling results for the no-mitigation case, otherwise referred to as the base case, to the basic reproduction number are shown in Supplementary Fig. 3. The rebound of the fraction of susceptible population indicates the effect of reinfection. Supplementary Fig. 4 shows the sensitivity of model results to other model parameters, such as the rate of reinfection and the randomness of the latent period and infectious period.

### Effects of non-pharmaceutical intervention measures

Before the vaccine and/or consensus on therapeutic treatment for COVID-19 become available or when the efficacy of existing vaccines is not high enough, NPIs are the only tool to combat the spread of the infection by lowering the peaks of daily new cases and deaths so that the health care infrastructure would not be overburdened. Please note that daily new cases refer to symptomatic infections only.

**Figure 2:**
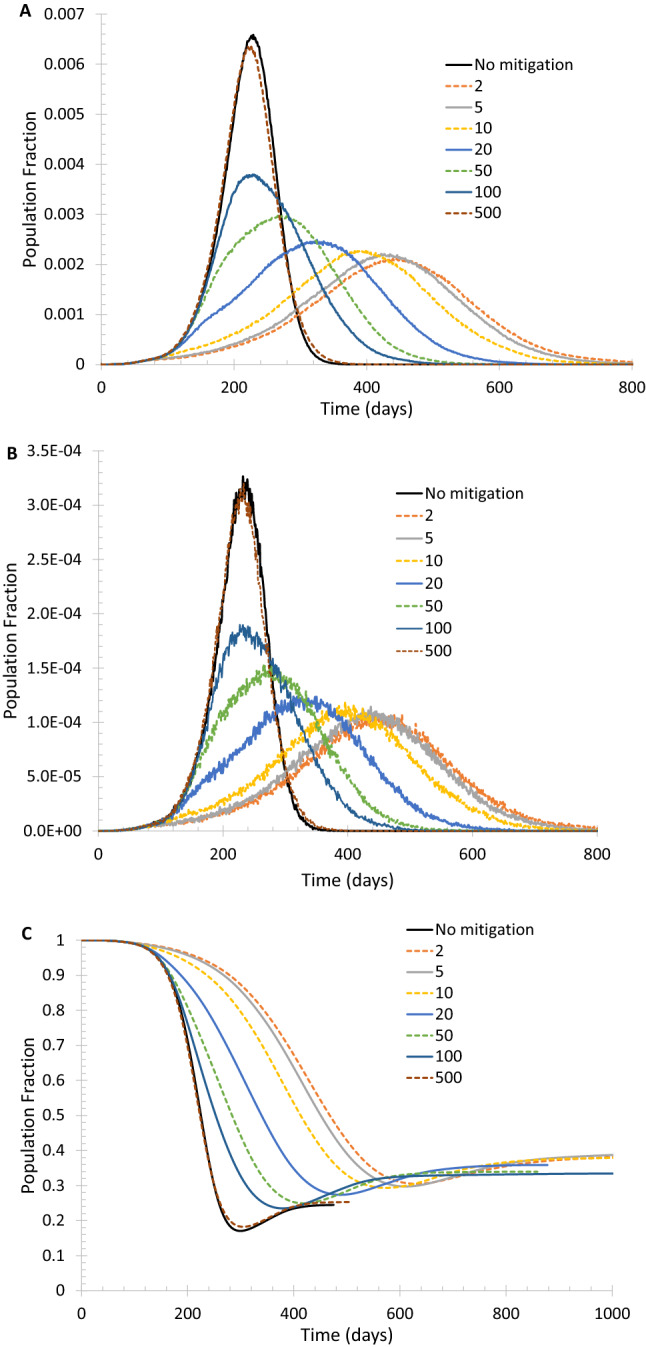
Delaying and lowering the peaks of daily new cases and daily new deaths by reducing the long-range random edges in the small-world network. Also shown is the evolution of the fraction of the susceptible population. In the calculation, the total number of random edges is reduced by 90%. (**A**) Daily new cases of symptomatically infected. (**B**) Daily new deaths. (**C**) Fraction of susceptible population.

An advantage of the small-world network is that the model can separately consider the effects of reducing community transmission and imported cases. Figure 2 shows that reducing long-range random edges by 90%, accomplished by substantially limiting international and domestic travel as well as strictly screening passengers at travel hubs, can indeed flatten the curve by delaying and lowering the peaks of daily new cases and daily new deaths. It is seen that, as expected intuitively, it is preferable to take the measure before the number of daily new cases exceeds 100 per 100,000 people, and the sooner the actions are taken, the more effective the NPIs are. If the measure is taken too late, e.g., when daily new cases reached 500 on a 100,000-vertex network, it will not make much of a difference. Supplementary Fig. 5 shows a slightly less stringent measure of reducing the long-range random edges by 80%, which is also helpful but the effectiveness in delaying and lowering the peaks is reduced considerably.

When to start mitigating community transmission can also affect how fast the infection spreads. Supplementary Fig. 6 shows that the timing of the lockdown doesn’t significantly change the eventual outcome, i.e., the final population fraction of susceptible as well as the total number of deaths and infected. Instead, when the peaks occur and how long it will take to attain herd immunity is heavily affected by the time at which the lockdown is triggered. Generally speaking, the sooner the lockdown starts, the longer it will take to reach the final state.

**Figure 3:**
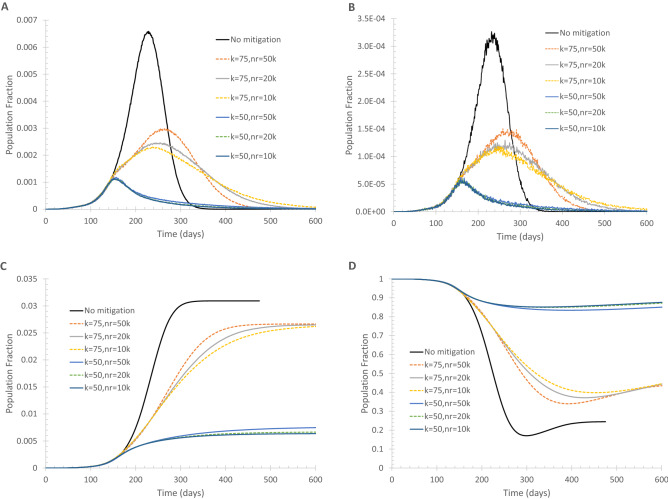
Effect of reducing both regular and long-range random edges on the spread of infection. Lockdown is triggered when daily new deaths = 10 and/or daily new cases = 200. (**A**) Daily new cases of symptomatically infected. (**B**) Daily new deaths. (**C**) Population fraction of total deaths. (**D**) Fraction of susceptible population.

Now, I show results for a case where lockdown is triggered when daily new deaths reach 10 and/or daily new cases reach 200. Reducing the number of regular edges each vertex has to its neighbors along with reducing long-range random edges can be more effective to lower and delay the peaks of daily new cases and deaths, as shown in Fig. 3. This is not surprising because the regular edges represent the everyday social interactions a person routinely has; eliminating some regular edges can lead to the reduction of the effective reproduction number R_e_. On the small-world network, the basic reproduction number R_0_ represents the number of vertices that can be infected by each infected vertex when each vertex is connected to k vertices by regular edges. When the number of regular edges is reduced to k_L_, the effective reproduction number is reduced to approximately R_0_k_L_/k. Supplementary Fig. 2 shows that reducing the basic reproduction number lowers the number of infections and deaths. The reduction of the number of regular edges each vertex has should not be taken literally. For example, the change from k = 100 to k = 50 does not mean each person actually cuts the number of people he/she interacts from 200 to 100, but rather, the reduction reflects the elimination of meaningful social interactions (i.e., long and close enough) that are capable of passing on the virus if one or more of the interacting persons is infectious. Supplementary Fig. 7 confirms that the benefit of reducing both the regular and long-range random edges together at a much earlier stage is merely to delay the peaks.

Two possible eventualities can be expected from implementing NPIs to reduce community transmission and imported cases. One is taking less restrictive measures to let the spread of infection continue but at a slower pace so that more time is gained to develop vaccine and therapeutic treatment, as shown by the k = 75 cases in Fig. 3. The other possibility, seen in Fig. 3 for the cases with k = 50, is to impose maximally possible restrictions so that the transmission of infection is kept minimal and lets it die out on itself. The benefit of the latter approach is obvious: fewer deaths and infection cases, but a drawback is that a new outbreak is always prone to occur because the susceptible population fraction remains high. With a high percentage of susceptible population, a few imported or asymptomatically infected cases can cause a resurgence of outbreaks. For a geographically isolated country or region like Iceland, New Zealand, and Taiwan, it may be possible, although still very difficult, to take the latter approach to limit imported cases once community transmissions are quenched. The development in New Zealand showed that infection resurgence could be real: after there were no new confirmed cases in late May and the first half of June 2020, new cases started to emerge in late June and the situation deteriorated in early August.

With a set of moderate mitigation measures, the peaks of daily new cases and daily new deaths can be reduced. In the meantime, more and more people will develop immunity, so even if the advent of widely available vaccines and therapeutics is delayed, the catastrophic resurgence of infection can be avoided.

Further, a prolonged lockdown may not be sustainable. This is evidenced by the reduction and rebound of on-road automobile traffic in three populous counties in California, USA as presented in Supplementary Fig. 8. The first statewide stay-at-home order along with other lockdown measures took effect around March 8, 2020 and remained uneased until May 18, 2020. However, the traffic rebound occurred several weeks before May 18, suggesting a significant portion of the population stopped following the lockdown orders and guidelines, i.e., the lockdown became less and less effective.

Before the lockdown decision is made, it is helpful to set a clear goal. If the purpose is to merely ‘flatten the curve’ to avoid collapsing the health care system and gain time for pharmaceutical interventions, the NPI measures can be implemented in a phased approach, i.e., adopt mitigation measures such as social distancing, tele-communicating and limiting the number of customers in stores. If those measures can achieve the anticipated result, then additional measures are not needed. On the other hand, if the mitigation measures do not seem as effective as expected, more restrictive measures should be developed and implemented. When evaluating the effectiveness of mitigation measures that have been in effect, it should be noted that it takes time to see the full impact of those measures. This argument is supported by the modeling results as presented in Fig. 3A. For the cases of reducing k from 100 to 75, the measures were implemented when the daily new cases surpassed 200 but still kept increasing until a turning point was reached. The reduced increase rate of daily new cases should be seen as a sign that the mitigation measures were working. Figure 3 further illustrates that if the purpose of mitigation measures is to stop the trend of the rapid growths of daily new cases and daily deaths, reducing k from 100 to 75 seems adequate. Of course, reducing k further to 50 can stop the growth more quickly, but the undesired outcome is that the susceptible population fraction remains high.

### Effect of reopening society

Once the lockdown has achieved the goal of lowering the peaks and when it is clear that the plague is unlikely to overwhelm the health care system, it is time to consider reopening society. This will have to be done in such a way that the infection won’t become out of control^29,30^. Intuitively, reopening may seem safe when both the daily new cases and daily new deaths are below certain reasonable thresholds for several consecutive days or when the day-by-day changes of the daily new cases and deaths are negative. However, these variables contain significant randomness. Even for an average of 100 realizations, as shown in Supplementary Fig. 9, the randomness is still noticeable. This means that sometimes, a consecutive drop of new cases and new deaths is merely a false signal of improvement.

A better threshold to be used to trigger reopening may be the fraction of susceptible population. The reasoning is straightforward: with fewer and fewer people remaining susceptible, the spread of the infection becomes less probable. For example, with R_0_ = 2.5, an infected individual can infect 2.5 persons when the entire population is susceptible; the number of persons that can be infected is reduced to 1.25 if only 50% of the population is susceptible even without any mitigation measures being implemented. The fraction of susceptible population can be estimated from population-based surveys of antibodies against SARS-CoV-2. Those surveys need the capability of mass antibody testing^31^.

**Figure 4:**
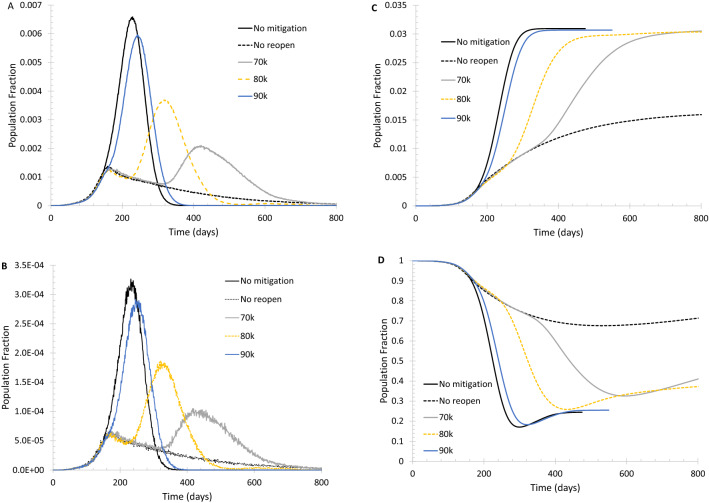
Spread of infection on a modulated small-world network. Initially, the network has its normal level of connections. When the number of daily new cases reaches 200 and/or the daily new deaths reaches 10, lockdown is implemented to reduce each vertex’s regular edges from 100 to 60 and the total number of random edges from 100,000 to 10,000. Full reopening (the number of edges is restored to the initial level) is triggered when the population ratio of susceptible is below a given threshold. (**A**) Daily new cases of symptomatically infected. (**B**) Daily new deaths. (**C**) Population fraction of total deaths. (**D**) Fraction of the population that remains susceptible.

As shown in Fig. 4, if lockdown to the level of k = 60 (from k = 100) and the total number of long-range random edges being 10,000 (from 100,000) is activated when daily new cases reach 200 and/or daily new deaths reach 10, and full reopening (k = 100 and p = 0.01 are restored) is initiated when the fraction of susceptible population is below 70%, the peak daily new cases and deaths can be reduced to about 1/3 of the base case level. A rebound of daily new cases and deaths seems inevitable unless reopening is started with a lower fraction of susceptible population. However, any delay to reopen will certainly prolong the entire duration of infection spread. A partial reopening (k = 75 and 50 000 long-range edges) can make the rebound a little weaker, as shown in Supplementary Fig. 10.

Supplementary Fig. 11 shows that the rebound of daily new cases and daily new deaths caused by a full restoration of random edges is relatively mild. This means that lifting travel bans may be administered sooner than fully reopening society. The reasoning is quite straightforward: once community transmission has started, the situation won’t deteriorate significantly by adding a few imported cases into the community.

## Concluding remarks

The results from the small-world network model provide insight into the spread of the infection and strategies to implement NPIs. As many countries and regions are contemplating to reopen society, the present findings can be employed to develop reopening plans. For countries and areas where the first wave of outbreaks has been successfully suppressed, this work provides useful information that is more reliable than the widely used continuous deterministic models to prepare for the potential resurgence of outbreaks.

This study is the first to report on modulating the spread of infection and identifying the optimal timing to impose and ease NPIs. Numbers of daily new cases and daily new deaths can be used to trigger NPIs, but they are not good indicators to decide reopening society when the NPIs seem to put the infection transmissions under control. Instead, the fraction of susceptible population is a reliable indicator for reopening. Further, if the purpose of implementing NPIs is to ‘flatten the curve,’ NPIs should be implemented in a measured pace so that the infection transmission cannot become out of control. On the other hand, if the purpose is to terminate the virus spreading, an all-out approach is more appropriate. However, the latter approach keeps the fraction of susceptible population at a high level, so outbreaks are prone to resurge.

## Supplementary Information

Supplementary Information.
