# Supplementary material for: Mitigating COVID-19 on a small-world network
Source: Sci Rep. 2021 Oct 14;11:20386. doi: 10.1038/s41598-021-99607-z (PMC8516975; doi:10.1038/s41598-021-99607-z)
Supplement: Supplementary file 1 — Supplementary Information. [file 41598_2021_99607_MOESM1_ESM.pdf]

Supplementary Information for

Mitigating COVID-19 on a Small-world Network

Marvin Du

Correspondence to: marvin.du@berkeley.edu

This PDF file includes:

Figs. S1 to S11

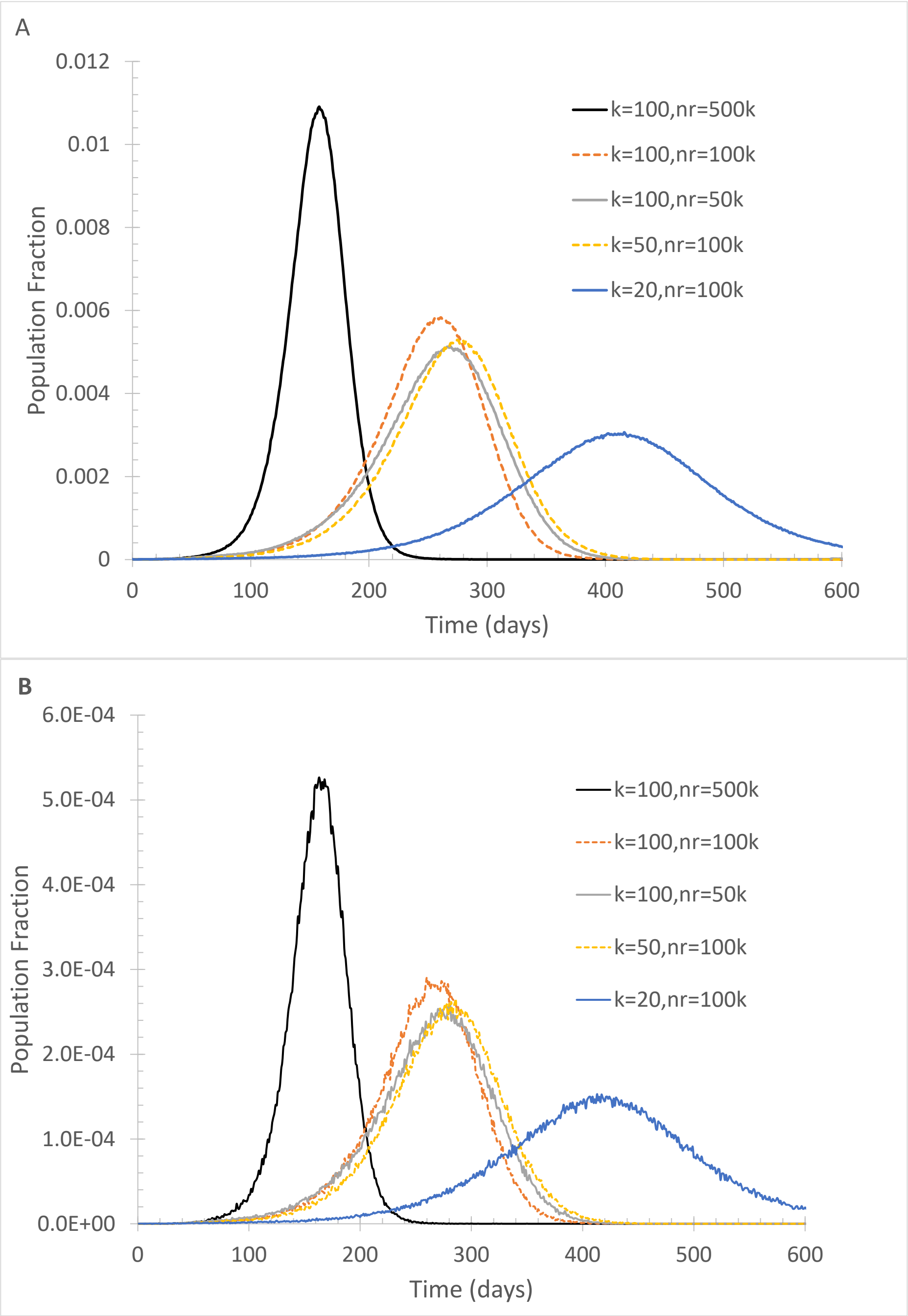

Fig. S1. Base case model results with different numbers of regular and long-range random edges. (**A**) Daily new cases of symptomatically infected. (**B**) Daily new deaths. (**C**) Population fraction of total deaths. (**D**) Fraction of susceptible population. In all the graphs, T_l = 3 days, T_i = 12 days, R_{asymp} = 0.3, R_0 = 2.5, and the probability of reinfection in 180 days is 0.2.

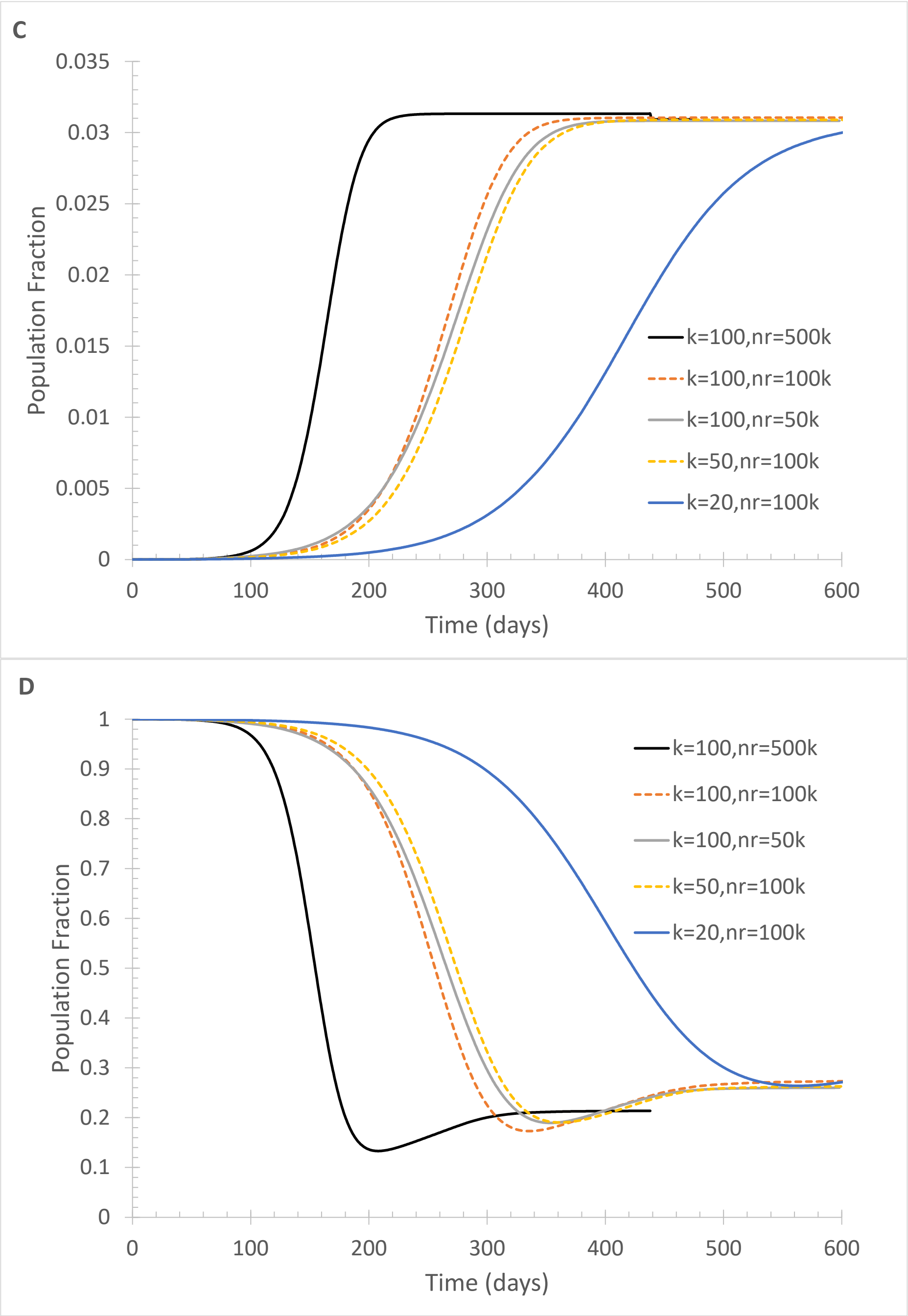

Fig. S1. (continued)

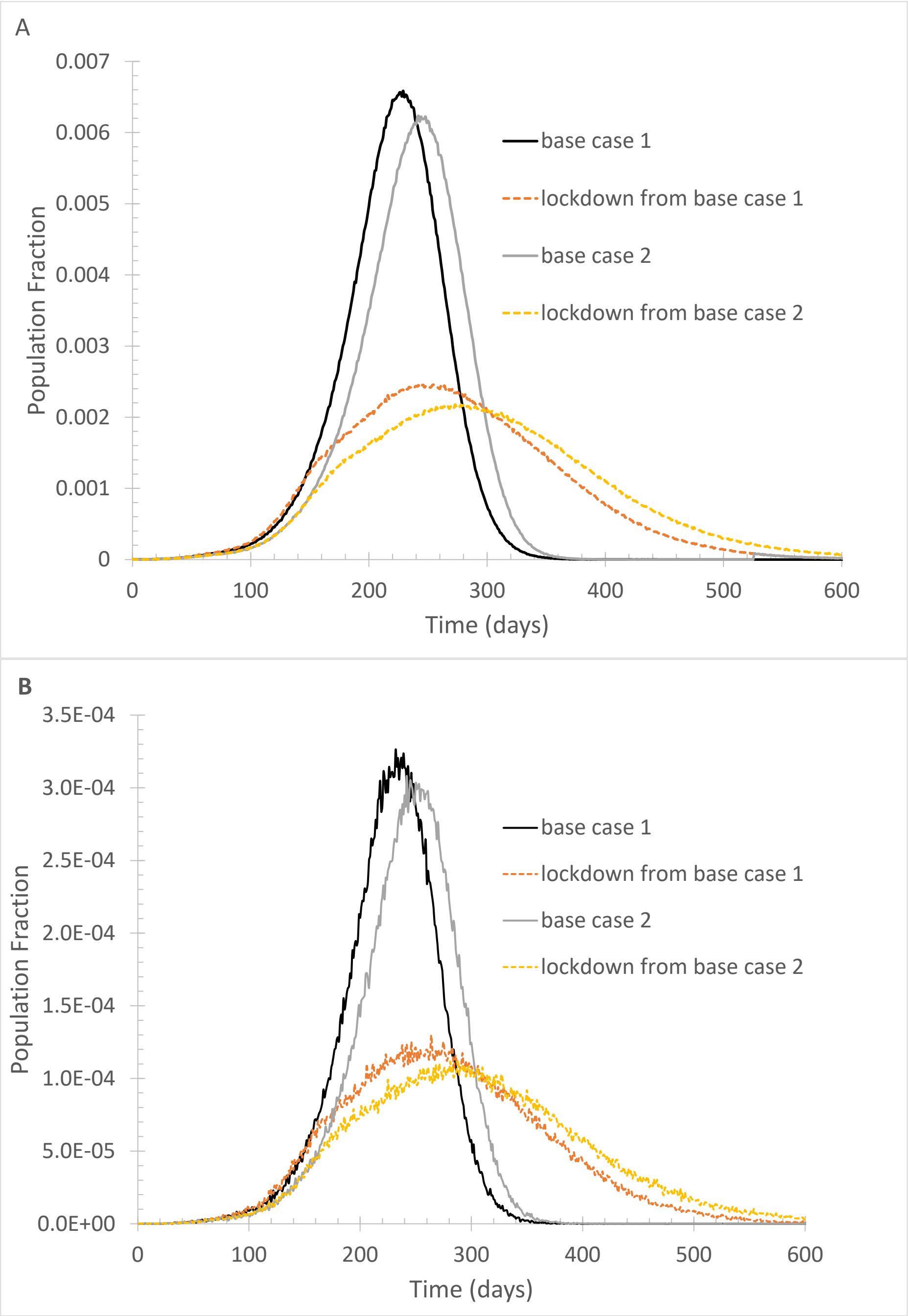

Fig. S2. Sensitivity test to examine the response to a 25% reduction of regular edges and 80% long-range of random edges. Base case 1: k = 100, p = 0.01; base case 2: k = 75, p = 0.01. Lockdown reduces k from 100 to 75 for case 1 and from k = 75 to 56 for case 2. (**A**) Daily new cases of symptomatically infected. (**B**) Daily new deaths. (**C**) Population fraction of total deaths. (**D**) Fraction of susceptible population. In all the graphs, $T_l = 3$ days, $T_i = 12$ days, $R_{asymp} = 0.3$, $R_0 = 2.5$, and the probability of reinfection in 180 days is 0.2.

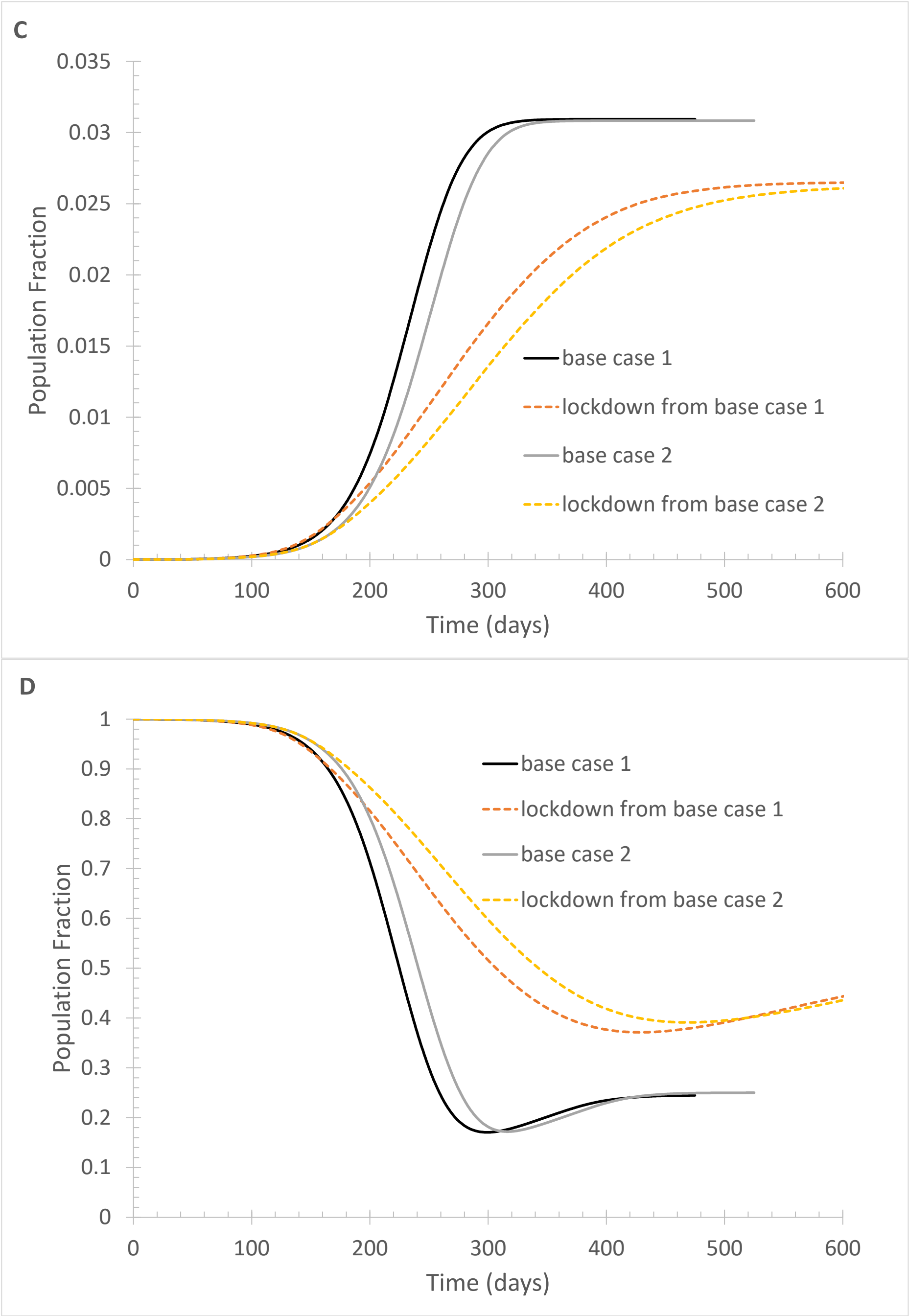

Fig. S2. (continued)

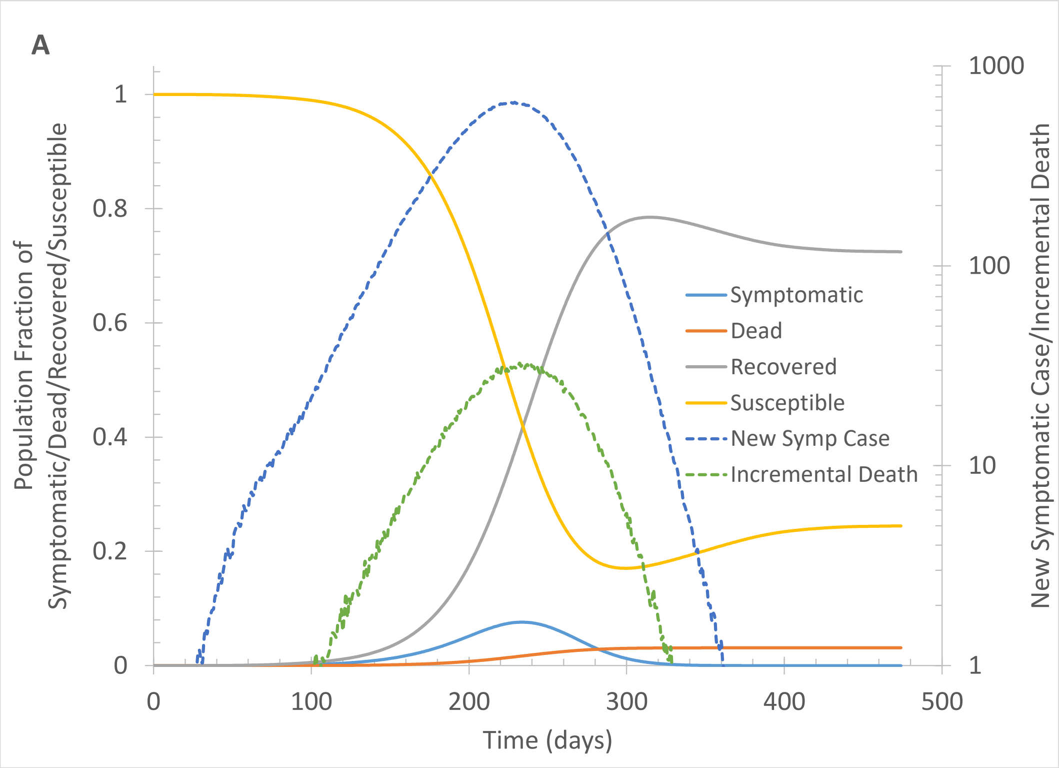

Fig. S3. An example of modeling results for the no-mitigation case. (A) Evolution of the compartments of symptomatically infected, dead, recovered, susceptible as well as daily new cases of symptomatically infected and daily new deaths. **(B)** Dependence of population fraction of susceptible on R_0. **(C)** Dependence of daily new cases of symptomatically infected on R_0. **(D)** Dependence of total deaths on R_0. In all the graphs, $T_l = 3$ days, $T_i = 12$ days, $R_{asymp} = 0.3$, and the probability of reinfection in 180 days is 0.2. In (A) $R_0 = 2.5$.

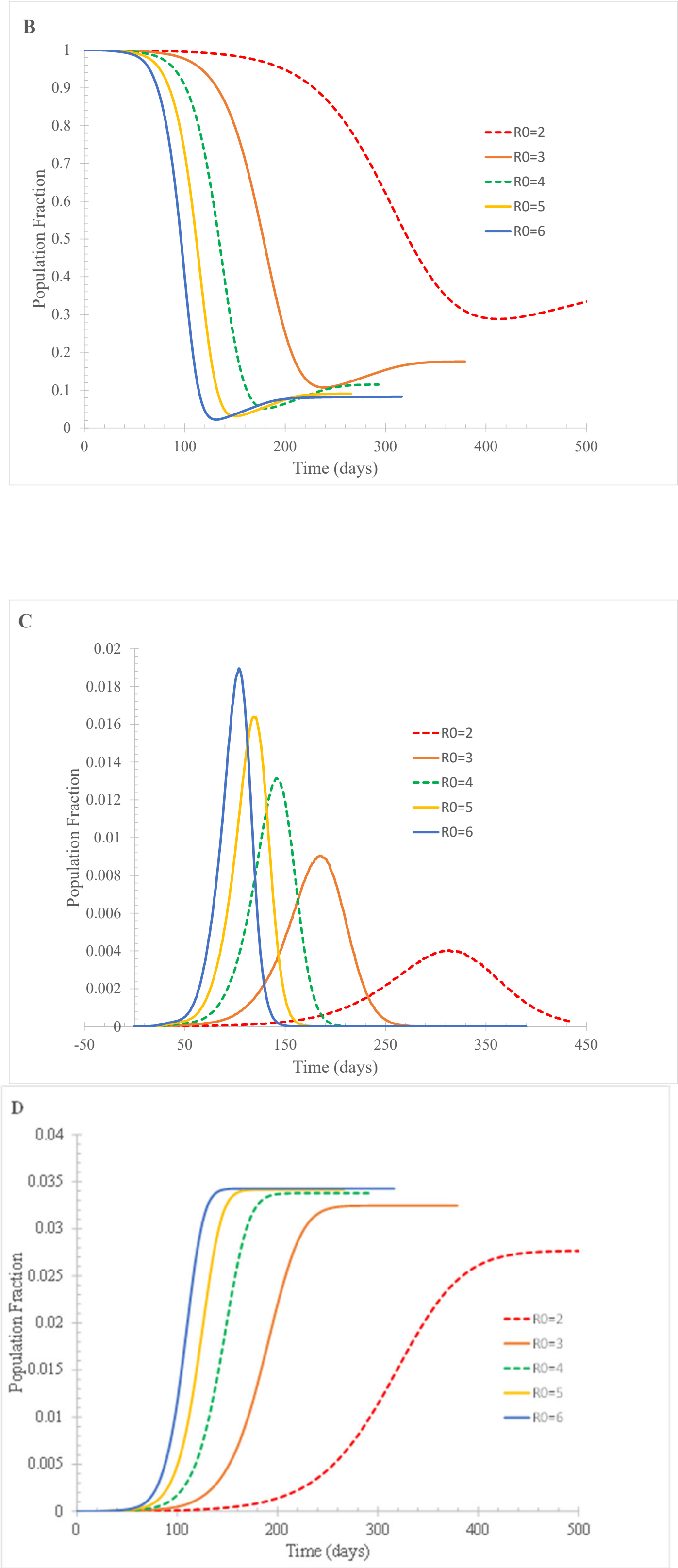

Fig. S3 (continued).

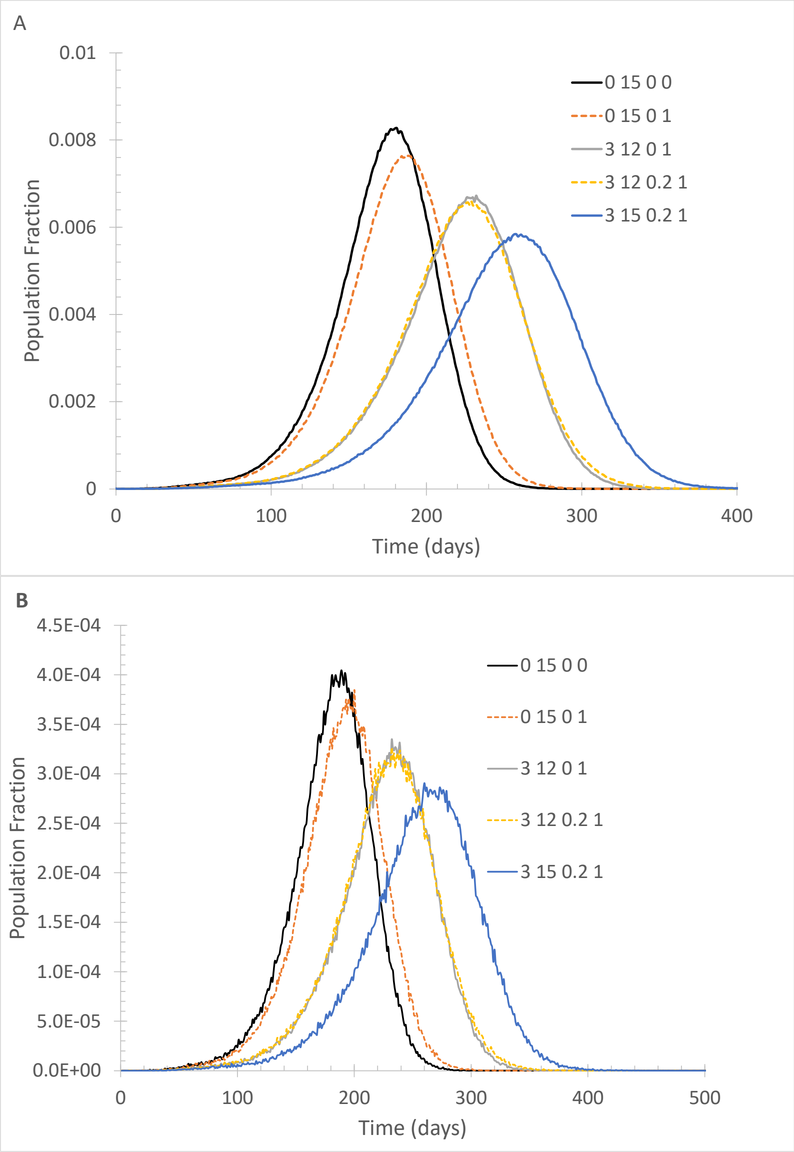

Fig. S4. Sensitivity of modeling results to model parameters. (**A**) Daily new cases of symptomatically infected. (**B**) Daily new deaths. **(C)** Population fraction of total deaths. (**D**)Fraction of susceptible population. example of modeling results for the no-mitigation case. The labels in the legend stand for latent period, infectious period, probability of reinfection in a period of 180 days, and randomness of those two periods where 1 means randomness is allowed and 0 means not allowed, and the rate of reinfection in a period of 180 days. In all graphs, R_0 = 2.5, R_{asymp} = 0.3, and R_{death} = 0.05, and the probability of reinfection in a period of 180 days is 0.2.

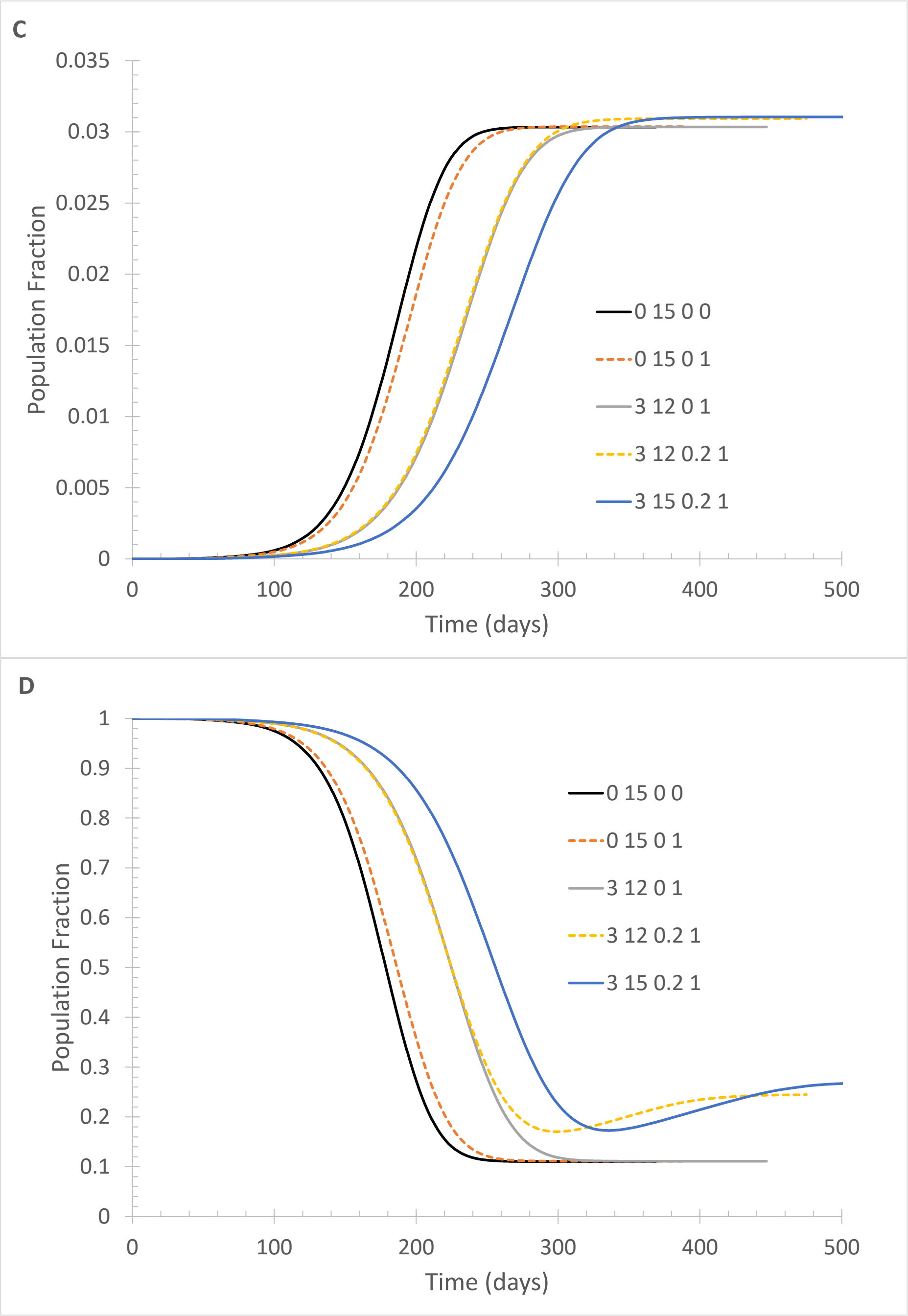

Fig. S4. (continued)

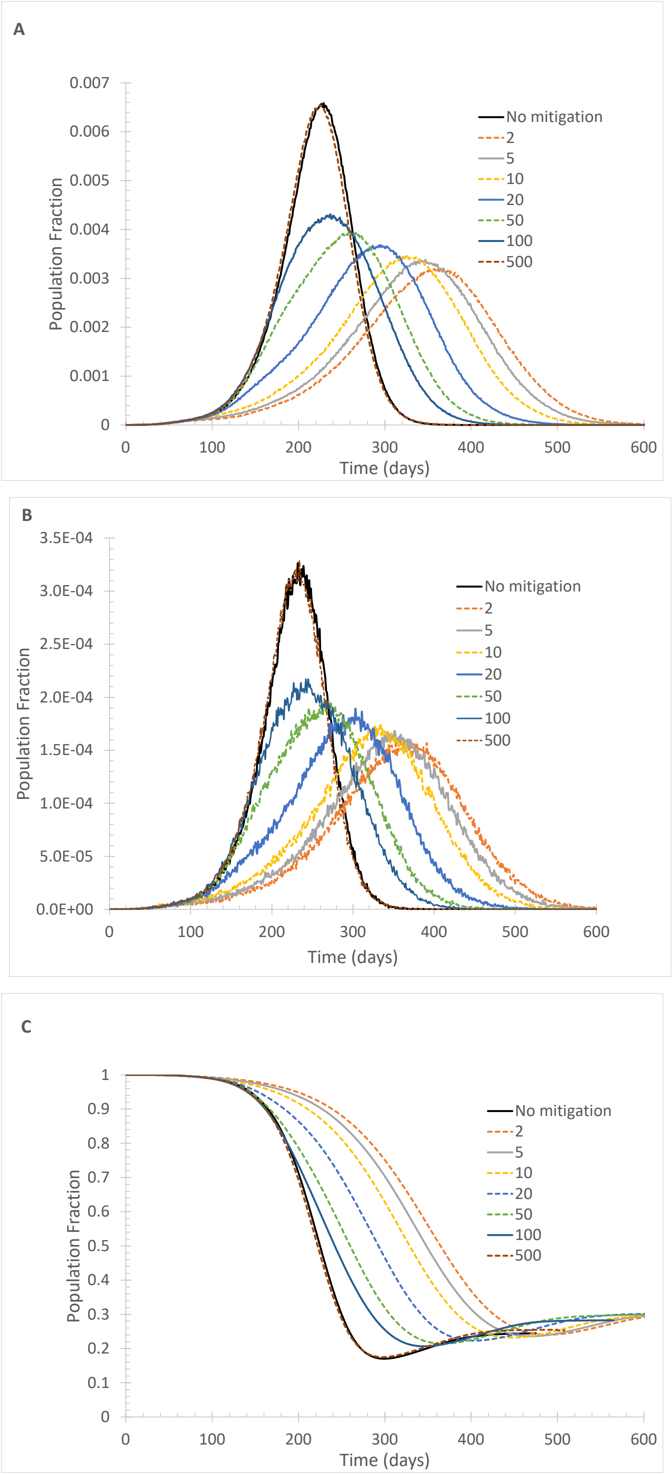

Fig. S5. Delaying and lowering the peaks of daily new cases and daily new deaths by reducing the long-range random edges in the small-world network. Also shown is the evolution of the fraction of the susceptible population. In the calculation, the total number of random edges is reduced by 80%. (**A**) Daily new cases of symptomatically infected. (**B**) Daily new deaths. (**C**) Fraction of susceptible population.

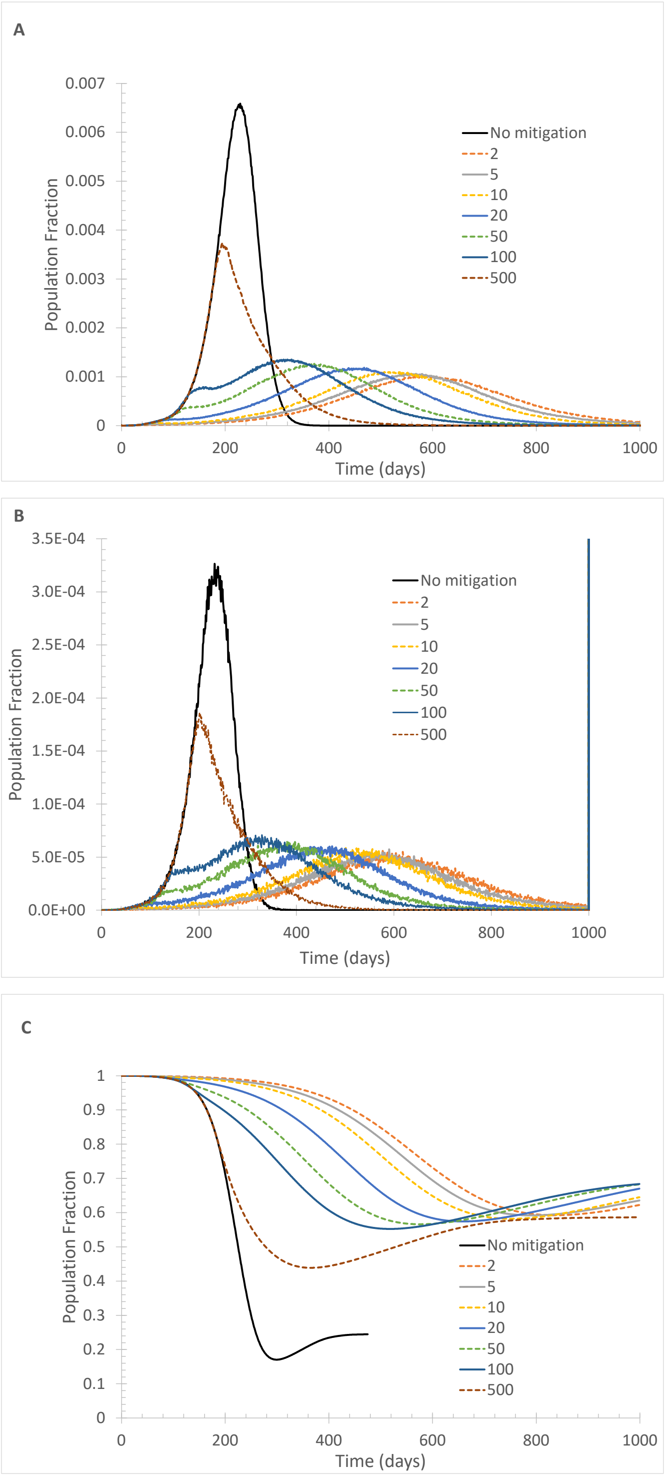

Fig. S6. Response of delaying and lowering the peaks of daily new cases and daily new death to the reduction of regular edges by 40%. Number of random edges is 100,000. The labels of the curves are the thresholds of daily new cases at which reduction of regular edges is started. Also shown is the evolution of the fraction of the susceptible population. (**A**) Daily new cases of symptomatically infected. (**B**) Daily new deaths. (**C**) Fraction of susceptible population.

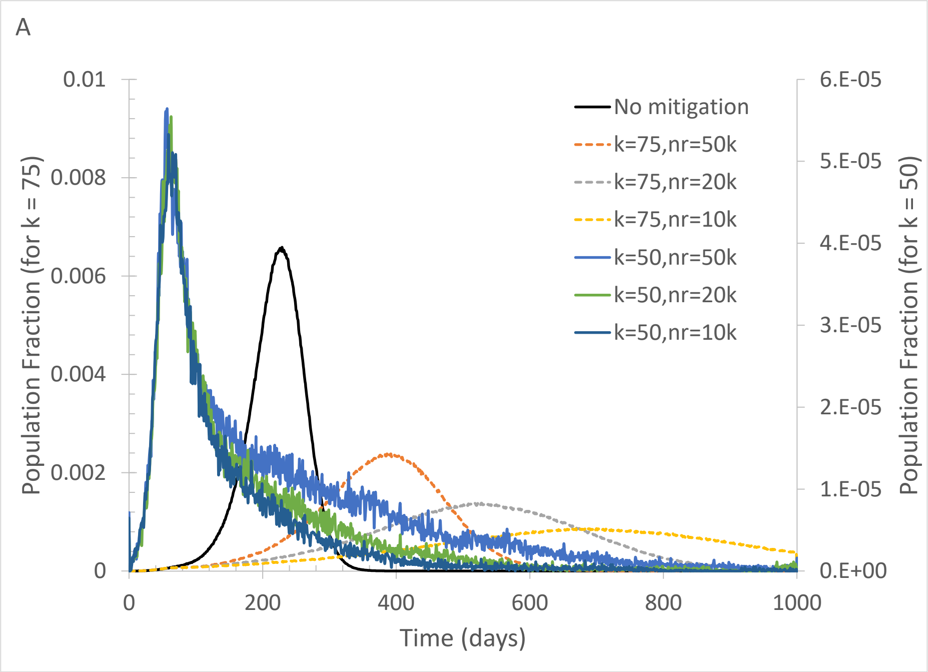

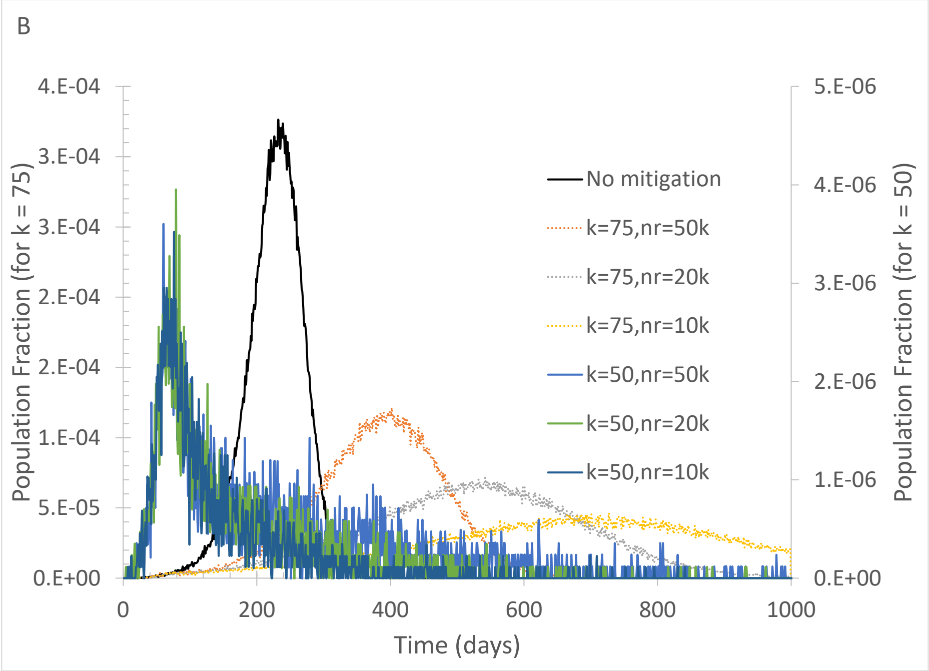

Fig. S7. Delaying and lowering the peaks of daily new cases and daily new deaths by reducing both the regular edges and the long-range random edges in the small-world network. Also shown is the evolution of the fraction of the susceptible population. Lockdown is triggered when daily new cases = 10. (**A**) Daily new cases of symptomatically infected. (**B**) Daily new deaths. (**C**) Population fraction of total deaths. (**D**) Fraction of susceptible population.

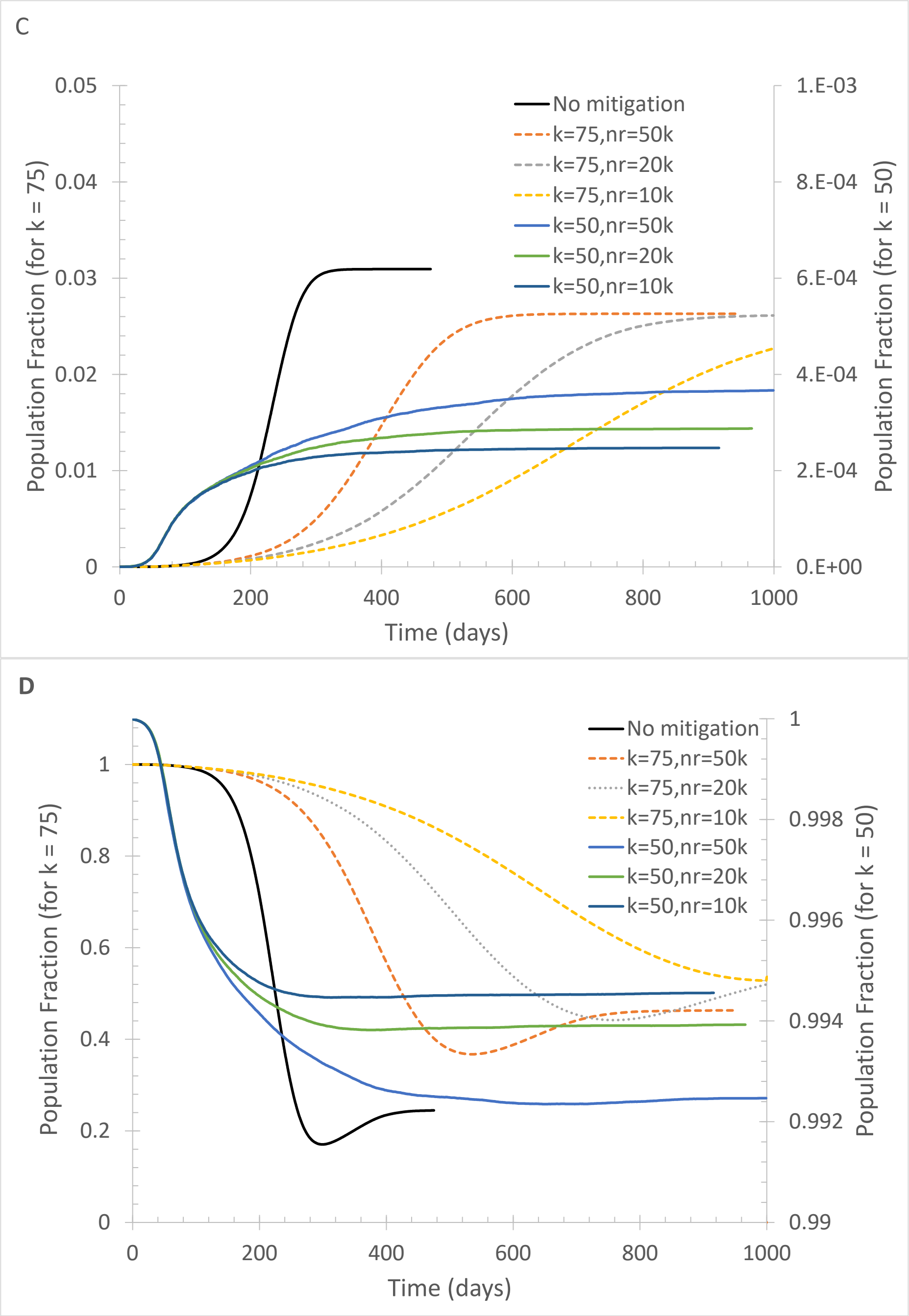

Fig. S7. (continued)

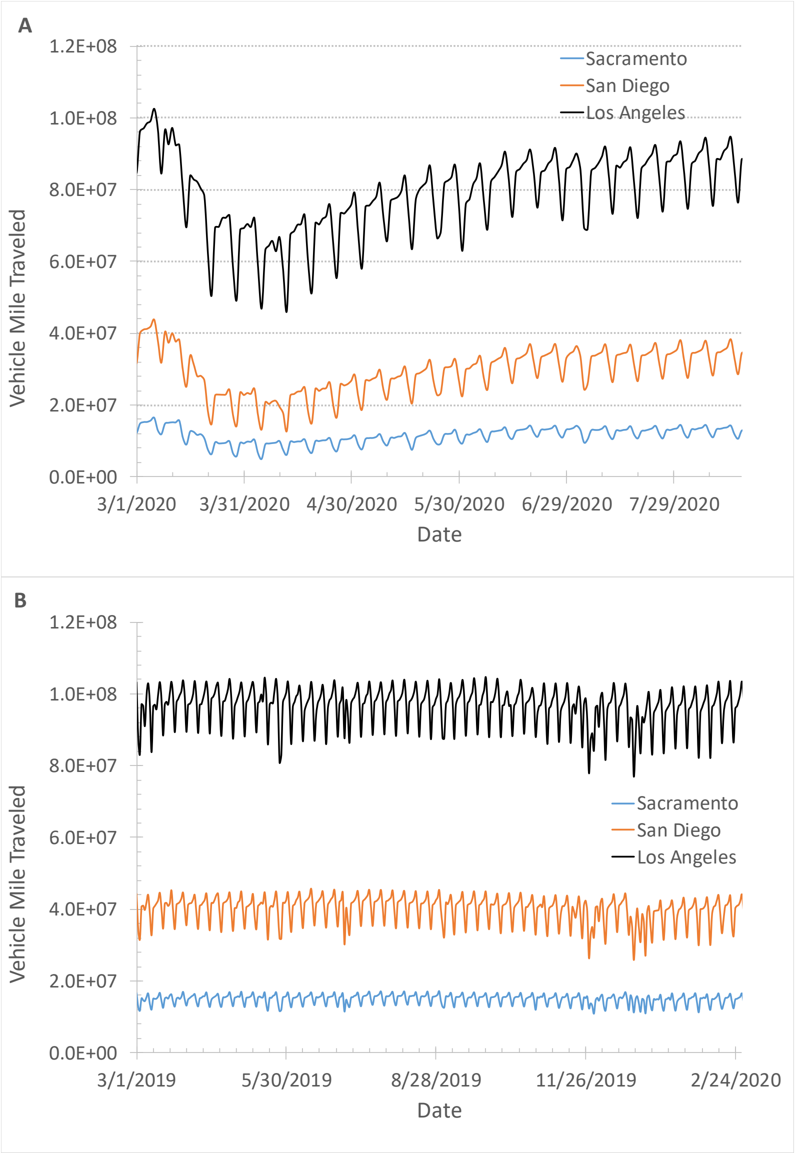

Fig. S8. Automobile traffic reduction on roads in three populous counties in California. (A) Vehicle miles traveled (VMT) from March 1, 2020 to August 18, 2020. **(B)** VMT from March 1, 2019 to February 29, 2020. It is clear that before the pandemic the on-road automobile traffic was steady and the day-to-day variation only reflected the day of the week, but significant reduction was seen to reflect the effect of the lockdown.

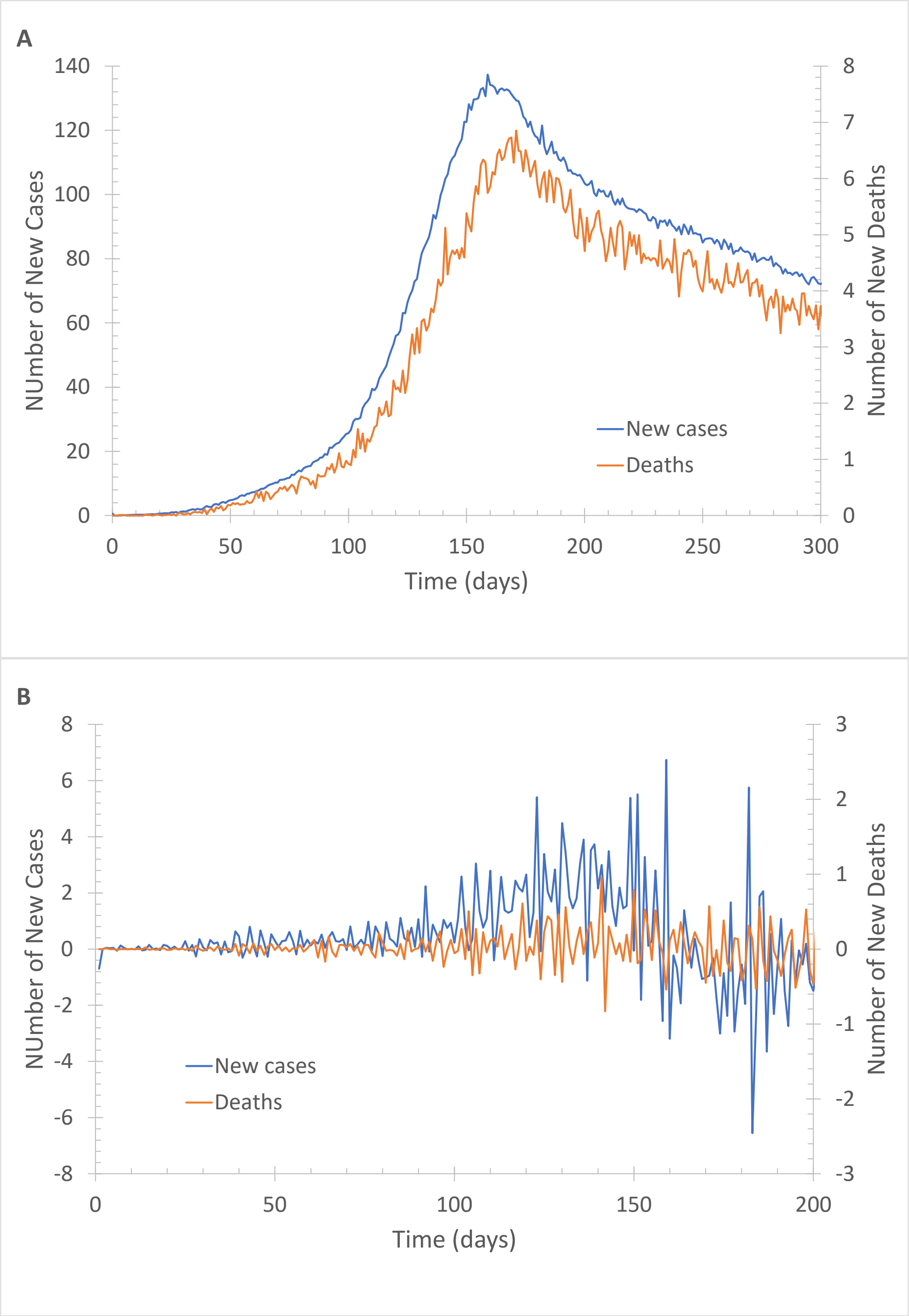

Fig. S9. A case study of lockdown to reduce each vertex's regular edges from k = 100 to k = 60 and total number of random edges from 100,000 to 10,000. All curves represent averaging over 100 realizations. (**A**) Daily new cases and daily new deaths. (**B**) Day-by-day variations of daily new cases and daily new deaths in a case study of lockdown.

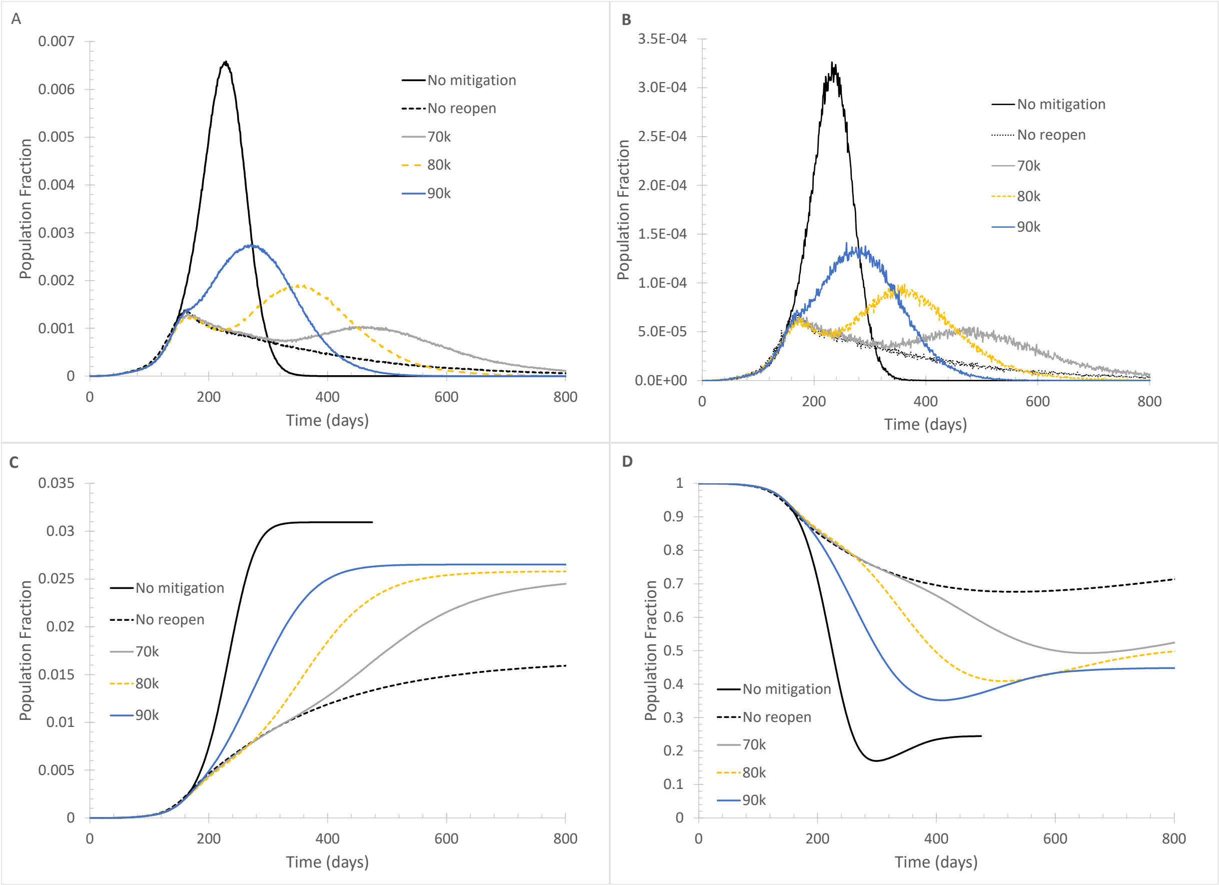

Fig. S10. Spread of infection on a modulated small-world network. Initially the network has its normal level connections. When the number of daily new cases reaches 200 and/or the daily new deaths reaches 10, lockdown is implemented to reduce each vertex's regular edges from k=100 to k=60 and the total number of random edges from 100,000 to 10,000. Partial reopening (the number of edges is restored to k = 75 and p = 0.005) is triggered when the population ratio of susceptible is below a given level of threshold. (**A**) Daily new cases of symptomatically infected. (**B**) Daily new deaths. (**C**) Population fraction of total deaths. (**D**) Fraction of the population that remains susceptible.

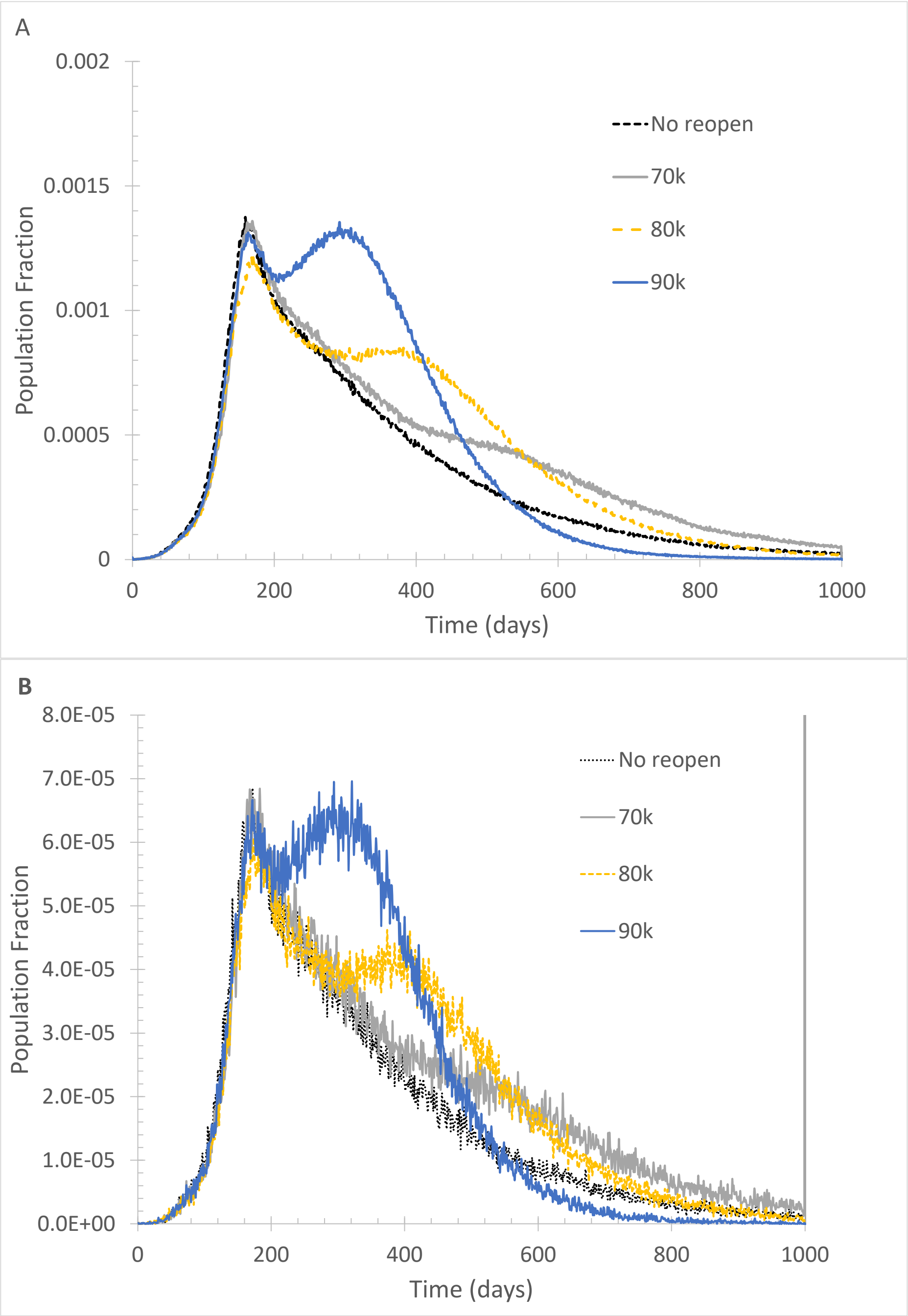

Fig. S11. The effect of restoring long-range random edges alone on daily new cases and daily new deaths. Initially the network has its normal level of connections. When the number of daily new cases reaches 200 and/or the daily new deaths reaches 10, lockdown is implemented to reduce each vertex's regular edges from k=100 to k=60 and the total number of random edges from 100,000 to 10,000. Partial reopening (the number of regular edges remained the same as in the lockdown mode, but the number of random edges is fully restored to 100,000). Reopening is triggered when the population ratio of susceptible is below a given threshold. (**A**) Daily new cases of symptomatically infected. (**B**) Daily new deaths.
